# Biofilms in the Food Industry: Health Aspects and Control Methods

**DOI:** 10.3389/fmicb.2018.00898

**Published:** 2018-05-07

**Authors:** Serena Galié, Coral García-Gutiérrez, Elisa M. Miguélez, Claudio J. Villar, Felipe Lombó

**Affiliations:** ^1^Research Group BIONUC (Biotechnology of Nutraceuticals and Bioactive Compounds), Departamento de Biología Funcional, Área de Microbiología, University of Oviedo, Oviedo, Spain; ^2^Instituto Universitario de Oncología del Principado de Asturias (IUOPA), Oviedo, Spain; ^3^Instituto de Investigación Sanitaria del Principado de Asturias (ISPA), Oviedo, Spain

**Keywords:** steel coating, quorum sensing inhibition, sanitizer, protease, bacteriophage, bacteriocin, biosurfactant, essential oil

## Abstract

Diverse microorganisms are able to grow on food matrixes and along food industry infrastructures. This growth may give rise to biofilms. This review summarizes, on the one hand, the current knowledge regarding the main bacterial species responsible for initial colonization, maturation and dispersal of food industry biofilms, as well as their associated health issues in dairy products, ready-to-eat foods and other food matrixes. These human pathogens include *Bacillus cereus* (which secretes toxins that can cause diarrhea and vomiting symptoms), *Escherichia coli* (which may include enterotoxigenic and even enterohemorrhagic strains), *Listeria monocytogenes* (a ubiquitous species in soil and water that can lead to abortion in pregnant women and other serious complications in children and the elderly), *Salmonella enterica* (which, when contaminating a food pipeline biofilm, may induce massive outbreaks and even death in children and elderly), and *Staphylococcus aureus* (known for its numerous enteric toxins). On the other hand, this review describes the currently available biofilm prevention and disruption methods in food factories, including steel surface modifications (such as nanoparticles with different metal oxides, nanocomposites, antimicrobial polymers, hydrogels or liposomes), cell-signaling inhibition strategies (such as lactic and citric acids), chemical treatments (such as ozone, quaternary ammonium compounds, NaOCl and other sanitizers), enzymatic disruption strategies (such as cellulases, proteases, glycosidases and DNAses), non-thermal plasma treatments, the use of bacteriophages (such as P100), bacteriocins (such us nisin), biosurfactants (such as lichenysin or surfactin) and plant essential oils (such as citral- or carvacrol-containing oils).

## Biofilms in the Food Industry

Biofilms are complex microbial ecosystems formed by one or more species immersed in an extracellular matrix of different compositions depending on the type of food manufacturing environment and the colonizing species. Examples of microorganisms that can comprise these biofilms include bacteria and fungi. The presence of more than one bacterial species in a biofilm has important ecological advantages because it can facilitate the biofilm’s attachment to a surface. For some species, this can even occur in the absence of specialized fimbriae. Mixed biofilms show higher resistance to disinfectants such as quaternary ammonium compounds and other biocides (Meyer, 2015).

The extracellular matrix is mainly composed of polysaccharides, such as cellulose, proteins or exogenous DNA. This matrix can be fixed to hard surfaces (food industry equipment, transport, dispensing and storage surfaces, soil, etc.) or to biological structures (vegetables, meat, bones, fruits, etc.) (Flemming et al., 2016). The extracellular matrix has a structural role, which is responsible for the strong persistence of these biofilms in the food industry. It generates complex gradients with respect to nutrients and oxygen diffusion, contains extracellular enzymes used for nutritional purposes, allows for the transfer of cell communication molecules, and protects the embedded cells against toxic compounds. In summary, biofilm formation confers many advantages to the microbial cells in a food industry environment, such as physical resistance (against desiccation), mechanical resistance (against liquid streams in pipelines) and chemical protection (against chemicals, antimicrobials and disinfectants used in the industry) (Flemming et al., 2016).

Biofilms can form quickly in food industry environments. The first two steps are the conditioning of the material’s surface and the reversible binding of the cells to that surface. Next, the binding becomes irreversible and the development of microcolonies begins. Finally, the biofilm’s tridimensional structure is formed, giving rise to a complex ecosystem ready for dispersion (Nikolaev and Plakunov, 2007; Srey et al., 2013; Coughlan et al., 2016).

Of particular importance to the food industry is that some biofilm-forming species in food factory environments are human pathogens. These pathogens are able to develop biofilm structures on different artificial substrates common in food industry, such as stainless steel, polyethylene, wood, glass, polypropylene, rubber, etc. (Abdallah et al., 2014; Colagiorgi et al., 2017).

Biofilm-associated effects (pathogenicity, corrosion of metal surfaces, alteration of organoleptic properties due to secretion of lipases or proteases) are of critical importance in some industries, such as dairy factories, where numerous processes and structures (raw milk tanks, pipelines, butter centrifuges, cheese tanks, pasteurizers, and packing tools) act as surface substrates for biofilm formation at different temperatures and with different colonizing species. For example, these biofilms may include the psychrotrophic *Pseudomonas* spp. and the thermophilic *Geobacillus stearothermophilus*. Fresh fish products may suffer from biofilm formation by pathogenic species (*Aeromonas hydrophila, L. monocytogenes, S. enterica* or *Vibrio* spp.), causing significant health and economic issues (Mizan et al., 2015).

Furthermore, biofilm-forming bacterial species can have genomic variations with respect to key genes involved in biofilm characteristics, giving rise to completely different biofilms under different conditions. This complexity, along with the high diversity of the affected environments and the variety of colonizing bacterial species, complicates biofilm eradication in the food industry.

## Health Aspects Associated With Food Industry Biofilms

Food-borne diseases associated with bacterial biofilms on food matrixes or factory equipment may arise via intoxications or infections. Toxins, for example, can be secreted by biofilm found within food processing plants. From there, they can contaminate a food matrix, causing individual or multiple (in the case of an outbreak) intoxications.

In either case, the presence of biofilms in a food factory puts human health at risk. The amount of risk is dependent on the bacterial species forming this tridimensional living structure. The main locations for biofilm development depend on the factory type, but may include water, milk and other liquid pipelines, pasteurizer plates, reverse osmosis membranes, tables, employee gloves, animal carcasses, contact surfaces, storage silos for raw materials and additives, dispensing tubing, packing material, etc. (Camargo et al., 2017).

The next sections describe the health and clinical aspects associated with the five most important food-borne bacterial pathogens, as well as their capacity to form biofilms on different surfaces.

### Bacillus cereus

*Bacillus cereus* is an anaerobic or facultative anaerobic Gram-positive and spore-forming bacterium that have the ability to grow over in different environments and in a wide range of temperatures (4–50°C) besides being resistant to heat, chemical treatments and radiation (Bottone, 2010). The persistence of vegetative forms of *B. cereus* in food processing surfaces has health importance. Also, this bacterium is able to survive industrial pasteurization processes due to the production of endospores. This fact complicates the removal of biofilm with cleaning procedures (Auger et al., 2009) and can affect biofilm persistence in dairy factories, reducing pasteurized milk and cream shelf-life, where levels of 10^3^ to 10^10^ CFUs have been detected in batches associated with outbreaks (Gopal et al., 2015).

Some strains of this bacterium are able to produce diarrheal enterotoxins, which cause diarrhea and abdominal pain (non-hemolytic enterotoxin NheA, cytotoxin K CytK, hemolysin BL HblC, cell wall peptidase EntFM), while other strains produce the emetic toxin (heat-stable cereulide), which causes vomiting symptoms. All of them are known to cause food poisoning. Of particular importance is the production of hemolysins by this common food-borne pathogen, which can result in extreme dehydration and even death (López et al., 2015; Tschiedel et al., 2015). The emetic syndrome caused by cereulide is related to the toxic activity of this small heat-stable peptide to the mitochondria. In this organelle, cereulide acts as a potassium ionophore, causing a cellular damage and immunomodulatory effects (Soni et al., 2016). In the other type of clinical manifestations, *B. cereus*-related diarrhea include abdominal cramps and watery diarrhea 8–16 h after ingestion. In this case, between 10^5^ and 10^8^ CFU are needed as infective dose (Soni et al., 2016).

*Bacillus cereus* biofilms are often associated with other microorganisms along food processing lines (Majed et al., 2016). This association is favored by their complex matrix of exopolysaccharides, proteins and extracellular DNA, which are necessary for its adhesion on different surfaces like glass (Vilain et al., 2009). The initial attachment of *B. cereus* on food manufacturing surfaces causes a preconditioning effect, since it facilitates the fast attachment of other bacterial species that would otherwise be removed by water flow, milk streams or other physical mechanisms present in these industries (Marchand et al., 2012).

This bacterial species is commonly found in dairy factories and in food and beverage plants (Ehling-Schulz et al., 2015; Ruan et al., 2015). In these dairy factories, *B. cereus* biofilms are found mainly at the air-liquid interface with a typical ring attached to the deposit wall from which the bacterial biofilm matrix protrudes onto the liquid surface (Fagerlund et al., 2014). However, some strains of this bacterium are also able to develop biofilms on submerged surfaces, for example, on stainless steel tanks and pipes (Wijman et al., 2007; Hayrapetyan et al., 2015a). Both at the air-liquid interface and under submerged conditions, flagellar motility is involved in biofilm development (Hayrapetyan et al., 2015b). From these biofilms, bacteria can easily migrate long distances along the food factory pipeline, posing serious health risks if they reach the food batches distributed to consumers (Hayrapetyan et al., 2015a). Of note, some *B. cereus* strains require milk components, such as natural surfactants and phospholipids, to colonize stainless steel equipment (Shaheen et al., 2010).

A study conducted in a plant producing pasteurized milk in Canada revealed that more than 5.5% of these products contained 10^5^ CFU/mL *B. cereus* and about 4% of these products contained enterotoxins at a level that may result in foodborne illness. The enterotoxin production by *B. cereus* in this pasteurized milk could occur in only 7–8 days of storage. These higher *B. cereus* counts were present in products with high butterfat content or in those ones processed with high-temperature, short-time pasteurization treatments (Saleh-Lakha et al., 2017). Also, traditional indicators, such as aerobic colony counts and psychotropic counts, showed no correlation with *B. cereus* levels in milk. 17 *B. cereus* isolates were characterized in a study from pasteurized milk. Five toxigenic gene patterns were identified in these isolates, all of them carrying genes coding for diarrheal toxins. Also, one strain contained all four diarrheal enterotoxin genes (Saleh-Lakha et al., 2017). This study reveled the importance of monitoring programs from food-borne pathogens, even in high-temperature pasteurized products. Another risk assessment for *B. cereus* in dairy industry compared ultrahigh-temperature and pasteurized milk collected at different supermarket chains in Brazil. Microbiology analysis revealed the presence of *B. cereus* in 33% of all analyzed manufacturers, where bacterial levels ranged from 10 to 10^3^ CFU/mL (Chaves et al., 2017).

The importance of this pathogen is highlighted by the fact that between 2007 and 2014, 6,657 persons were reported to suffer food-borne intoxications due to *B. cereus* in the EU (505 of them just in 2014), associated to 413 outbreaks. No casualties were reported. The involved food matrixes in these outbreaks were buffet meals (27.6%) cereal products (10.9%), red meat products (8%), poultry meat products (5.3%) and vegetables or juices (4.6%) (EFSA Panel on Biological Hazards [BIOHAZ], 2016). Regarding the other pathogens included in this review, *B. cereus* ranks after *Salmonella enterica* (26.2% of all cases), *S. aureus* food poisoning (9.3%), enterohemorrhagic *E. coli* (1.7%) and *L. monocytogenes* (0.7%) (EFSA Panel on Biological Hazards [BIOHAZ], 2016; European Food Safety Authority European Centre for Disease Prevention and Control, 2017).

### Enterohemorrhagic *Escherichia coli*

Most *E. coli* strains are part of human intestinal microbiota, where they do not represent a health problem. However, other strains do pose a health risk and are noxious foodborne pathogens transmitted by drinking water, fruits and vegetables (tomatoes, melons, parsley, cilantro, lettuce, spinach, etc.), raw milk or fresh meat. These products may have been contaminated at their origin or as part of the food manufacturing process. In the food industry, this contamination may take place during the pre-harvest period, due to the use of a contaminated water supply when cultivating the vegetables. This contamination may also take place in post-harvest environments, where it may appear after washing and processing the raw material (carcasses, vegetables, etc.), but also due to storage temperatures which allow fast growth of the present bacterial contaminants (Carter et al., 2016).

Many studies have demonstrated that *E. coli* strains can attach to a variety of surfaces including stainless steel, Teflon, glass, polystyrene, polypropilene, PVC and biotic surfaces. The hydrophobicity of the surface material plays an important role in biofilm formation by this species. For example, *E. coli* O157:H7 strain showed strong biofilm formation on borosilicate glass and stainless steel, but little or no biofilm was observed on polypropylene, probably due to its hydrophobic nature (Carter et al., 2016). For example, The initial attachment of *E. coli* O157:H7 and biofilm development on the surfaces is enhanced by the presence of flagella and fimbria (type 1 or curli) (Van Houdt and Michiels, 2010).

Temperature is another important factor that affects *E. coli* biofilm formation. For example, when *E. coli* O157:H7 was incubated on beef surfaces at 15°C for 7 days, the number of adherent and planktonic cells increased (Dourou et al., 2011). This behavior represents a serious issue for meat processing plants, where the regular working temperature is 15°C.

Similar to *B. cereus, E. coli* O157:H7 biofilm formation may include other species, resulting in emergent ecological benefits. For example, survival rates of this strain on food industry surfaces were enhanced up to sixfold in the presence of other species such as *Ralstonia insidiosa* or *Burkholderia caryophylli* (Liu et al., 2014).

*Escherichia coli* survival under stress conditions and its biofilm formation abilities are serotype-dependent. For example, the serotype O157:H7 (a common STEC strain) displayed a high resistance to temperature, high pressure and common food industry disinfectants when compared to other pathogenic and non-pathogenic *E. coli* strains, such as O111:H-, O103:H25, O26 or O145 (Álvarez-Ordóñez et al., 2013; Chagnot et al., 2014).

*Escherichia coli* is also well known because of their acid resistance mechanisms, which is an important factor in the case of pathogenic strains such as O26 (an enterohemorrhagic strain), which allow this bacterium to survive in extreme acid conditions, as those generated in some food industry processes involving acetic (commonly used in some canned vegetable products), citric (commonly used in fruit juice industry as a preservative), as well as propionic and lactic acids (commonly used in fermented dairy and meat products) (Lajhar et al., 2017). These acid resistance mechanisms have been studied in the *E. coli* MG1655 strain (a derivative of the non-enterohemorrhagic K-12 strain), where they are directly regulated by *nac* and *csiR* genes, which code for a transcriptional regulator affecting genes involved in nitrogen metabolism and for a repressor acting on TCA (tricarboxylic acid) cycle reactions, respectively (Aquino et al., 2017). This fact is of high importance with respect to health issues, as surviving enterohemorrhagic *E. coli* O157:H- and O145 cells stored in acidic food matrixes were more resistant to gastric acid challenge and therefore a lower infective dose is needed (McLeod et al., 2016).

Regarding clinical issues, Shiga toxin (Stx)-producing *E. coli* (STEC) secrete Stx1 and Stx2 toxins and cause enterohemorrhagic gastroenteritis, which presents with watery diarrhea and blood in the feces. Soups, sauces, cooked chicken, ground beef, salads and other fresh products contaminated with this strain have lead to outbreaks and even death (Yang et al., 2017). In some cases, this illness evolves in some cases toward hemolytic uremic syndrome, with acute kidney injury and thrombocytopenia (Carter et al., 2016; Bowen and Coward, 2017; Cha et al., 2018).

Another major problem of STEC strains, such as *E. coli* O157:H7, is that less than 50 ingested CFUs are necessary for an infectious dose. Therefore, even a low-grade biofilm contamination of a food factory installation is a serious health problem and requires that strong control measures be in place (Marouani-Gadri et al., 2010).

Between 2007 and 2013, in the EU, 423 foodborne outbreaks associated with STEC and other pathogenic *E. coli* were reported. The majority of cases were caused by the serotype O157, however, between 2011 and 2013 the main source of the cases was the serotype O104 (7% were caused by serogroup O127 and 9% were caused by unknown serotypes). The major outbreak of the serotype O104 was first identified in Germany in 2011 and it was epidemiologically associated with the consumption of fenugreek sprouts. This outbreak resulted in over 4,000 cases of infection and 54 deaths in 14 European countries, United States, and Canada (EFSA Panel on Biological Hazards [BIOHAZ], 2015). Also, 885 cases of haemolytic uremic syndrome (HUS) and 3,019 cases with diarrhea were reported as a consequence of this outbreak (European Food Safety Authority, 2011). Another case of HUS was reported in Romania and Italy in 2016, with an overall of 19 cases and 3 deaths. In this case, the majority of the cases were due to the serotype O26, and a possible source of infection was a milk processing establishment in Romania (European Food Safety Authority, 2016).

### Listeria monocytogenes

The Gram-positive bacterium *L. monocytogenes* is a ubiquitous, dangerous, foodborne pathogen. However, it is not resistant to pasteurization treatments (Milillo et al., 2012). Some examples of food products known to transmit this pathogen are seafood, dairy products, meat, ready-to-eat products, fruits, soft cheeses, ice cream, unpasteurized milk, candied apples, frozen vegetables, and poultry (CDC, 2017; Rothrock et al., 2017).

*Listeria monocytogenes* biofilms are mainly composed of teichoic acids and can grow on polypropylene, steel, rubber or glass surfaces throughout the industry. From there, this pathogen spreads to food batches, where it can replicate at refrigeration temperatures (Silva et al., 2008). Common contaminated foods are smoked fish, cold cuts and fresh cheese. Together with this low temperature replication ability, this bacterium enhances its hydrophilicity and induces biofilm status as a response to cold temperatures, increasing its attachment to surfaces and its resistance to cleaning procedures in many food factories (Zhang et al., 2016; Lee et al., 2017). The eradication of this pathogen in the food industry is further complicated by its resistance to treatments up to 60°C (Møretrø et al., 2017). *L. monocytogenes* genes involved in flagellar motility (*fliQ, flaA, fli1, motA*) are necessary for biofilm formation, as are the regulatory gene *phoR* (from the phosphate sensing operon) and the genes involved in D-alanine incorporation into lipoteichoic acid (Alonso et al., 2014).

This pathogen causes gastroenteritis in healthy individuals. In pregnant women, infants, the elderly and immunocompromised individuals, this bacterium causes listeriosis, a critical disease which also involves septicemia and meningitis (Silk et al., 2012). In pregnant women, listeriosis can lead to spontaneous abortion or damage to the fetus (Ferreira et al., 2014). Following ingestion, after host cell invasion, *L. monocytogenes* can use listeriolysin O (LLO) and/or phospholipases PlcA and PlcB, to get into the cytosol of the human cell (Kanki et al., 2018). LLO is a cholesterol-dependent cytolysin that inserts into host cellular membranes, forming pores and breaking vacuolar compartments acceding to the cytosolic space. Then, the bacterium uses the surface protein actin-assembly inducing protein (ActA) to activate the actin-assembly machinery of the host cell, in order to facilitate intracellular bacterial movement and cell-to-cell spread (Kanki et al., 2018).

Along 2016, 2,536 confirmed human cases of listeriosis have been reported, showing a continuous increase since 2012 (1,720 confirmed human cases) (European Food Safety Authority, 2016). Despite only five reported outbreaks due to this pathogen in 2016, it has been linked with the highest proportion (8.0%) of deaths among illness in that year (European Food Safety Authority, 2016). In a similar way, four listeriosis outbreaks, spanning from 2014 through 2016, have been linked to *L. monocytogenes* serotype 4b variant (4bV) strains (Burall et al., 2017). The high degree of relatedness among these 4bV strains after genetic analysis has led the authors to suggest the possibility of cross-contamination between the involved facilities. An important reason for this cross-contamination events in cheese, salmon, meat or other food matrixes industries is the existence of persistent *L. monocytogenes* strains, which show better adherence to stainless steel surfaces, resistance to quaternary ammonium compounds or benzalkonium chloride (after sublethal exposition), resistance to pH or temperature, or higher invasiveness of eukaryotic cells (Ferreira et al., 2014; Martínez-Suárez et al., 2016). These factors highlight the great clinical importance of *L. monocytogenes* biofilms monitoring and control in food industry.

### Salmonella enterica

This foodborne pathogen causes gastroenteritis or septicemia (in the case of some serovars) (Wang et al., 2013b). *S. enterica* serovar Enteritidis is the most frequent serotype generating nausea, vomiting, fever, diarrhea and abdominal pain as main symptoms (Nguyen et al., 2014). Poultry meat is a common reservoir for these bacteria in processed food. Its importance as a food pathogen is demonstrated by the fact that *S. enterica* biofilm formation on food surfaces was the first reported of these complex, multicellular structures (Duguid et al., 1966). *S. enterica* is able to grow on stainless steel surfaces, resulting in a 3D structure with several layers of cells, which may present different morphologies depending on the available nutrients. For example, an areticulum-shape was generated when cultured in tryptic soy broth (TSB) medium (Wang et al., 2013a,b). The topography influences its initial attachment to a surface. For example, untreated and mechanically sanded steel was the most easily colonized metal. However, electro-polished and bright-alum finished steel were poorly colonized by this bacterium (Schlisselberg and Yaron, 2013). Nonetheless, in contrast to other pathogens described above, glass surfaces were not a suitable material for *S. enterica* biofilm production (De Oliveira et al., 2014). Of particular importance is that under dry conditions *S. enterica* can survive in a biofilm on stainless steel for over a year. From there, it is possible for this bacterium to contaminate thousands of food batches (Morita et al., 2011).

As with other Gram-negative bacilli, the cell envelope of this pathogen contains lipopolysaccharide, which functions as an endotoxin, and is important as virulence factor: it evokes fever, activate the serum complement, kinin, and clotting systems, depress myocardial function, and alter lymphocyte function. Therefore, circulating endotoxin may be responsible in part for many of the manifestations of septic shock that can occur in systemic infections by *Salmonella* (Hagar et al., 2013). Also, this bacterium triggers endocytosis in the M cells of the intestinal mucosa, and is able to inject the AvrA toxin via a type III secretion system, inhibiting the innate immune response of the host (Haraga et al., 2008).

*Salmonella enterica* is capable of attaching to meat and other food matrixes easily, eventually leading to cross-contamination between food batches in a manufacturing plant or supermarket, a fact that further underscores the serious health concern this bacterium poses with respect to outbreaks risk, for example associated to refrigerated poultry products in shelves during food processing or sale in a supermarket (Wang et al., 2013a). In fact, the main source of contamination by this bacterium is biofilm formation in infrastructures used during pre-cooked foods manufacturing (such as pre-cooked chicken), a process that has given rise to outbreaks affecting thousands (over 2,000 in Spain in 2005) of people and can sometimes be lethal (Lenglet and National Epidemiological Surveillance Network of Spain, 2005; Wang et al., 2013b). In the case of systemic infections, these fatalities can reach up to 20% of the affected patients during an outbreak, especially children and immunocompromised individuals (Mahon and Fields, 2016). In 2013 and 2014, an outbreak caused by contaminated pork sausages in Germany affected 145 elderly people (Simon et al., 2018).

In 2016, *S. enterica* was identified as the second most frequently agent (just after *Campylobacter*) of food-borne and water-borne outbreaks in the EU, with 94,625 cases. This bacteria accounted that year for 12,353 hospitalizations and 126 deaths (50% of all deaths associated to outbreaks) (European Food Safety Authority, 2016). These numbers show a reduction with respect to previous decade, mainly due to more strict control and detection measures at production factories and distribution chains.

### Staphylococcus aureus

*Staphylococcus aureus* is a Gram-positive, non-spore forming, non-motile, facultative anaerobic bacterium. It is a human opportunistic pathogen, largely due to its characteristic production of enterotoxins at temperatures between 10 and 46°C. This species is able to multiply on the mucous membranes and skin of food handlers, a major issue for food factories (Giaouris et al., 2015), because staphylococcal enterotoxins are heat-stable and are secreted during growth of this bacterium in a food matrix, eventually contaminated by the food handler or an animal. Food matrixes with a low water activity, such as those with high sugar or salt content, are suitable for this bacterium. These enterotoxins bind to class II MHC in T cells, giving rise to their activation and to an acute toxic shock with diarrhea and vomiting (Schelin et al., 2017).

Moreover, the emergence of methicillin-resistant *S. aureus* (MRSA) in farm animals has caused great concern because animal-derived foods are a primary contamination origin for this resistant pathogen and this bacterium is able to form biofilms on many different kinds of animal surfaces (Vergara et al., 2017). *S. aureus* can form biofilms on both biotic and abiotic surfaces along the food production chain. This factor carries considerable economic importance since the removal treatment is different depending on the matrix composition. Some options for this removal are glycoside hydrolases (such as Dispersin B, produced by *Aggregatibacter actinomycetemcomitans*) and proteases (such as proteinase K, a serine protease from the fungus *Engyodontium album*) (Kaplan, 2010; Fagerlund et al., 2016).

The growth of *S. aureus* biofilms is enhanced by various processing methods encountered in the food industry, such as suboptimal temperatures, improper disinfection or a combination of salt and glucose. The transcription of genes involved in biofilm formation and virulence in this pathogen (surface proteins, proteases, capsular polysaccharides) is upregulated in the presence of sub-lethal concentrations of various common detergents used in the food industry (Slany et al., 2017). The expression of the *icaA* gene, whose transcriptional product *N*-acetylglucosaminyltransferase is involved in the biosynthesis of an extracellular polysaccharide matrix, is a major factor in biofilm formation in this species (Abdallah et al., 2015). Other genes involved in the biofilm formation of this pathogen play a role in adhesion (*icaD, cna, fnbA* and *fnbB*), toxin secretion (*hla* and *hlb*) and transcriptional regulation (*agr* and *sarA*) (Vergara et al., 2017).

In 2015, 434 outbreaks were caused by this bacterium in the EU (EFSA Panel on Biological Hazards [BIOHAZ], 2015). In 2016, in the United States, 241,994 cases of this type of food poisoning were reported, with 1,067 hospitalizations and 6 deaths (CDC, 2016).

## Methods for Controlling Biofilm Formation in the Food Industry

The development of online monitoring methods to follow the adhesion, growth and/or removal of deposits and biofilms from surfaces at industrial environment reduces the cost of cleaning operations and minimizes production breaks for maintenance. Classical methods for biofilm detection, such as agar plating, are not effective due to the difficulty in culturing many biofilm bacteria. This is due to the fact that some foodborne pathogens, such as *L. monocytogenes*, can enter into a ‘viable but non-culturable’ (VBNC) form with low metabolic activity. These VBNC cells cannot be detected by culture methods and may even lead to the survival of cells under stress conditions, such as low temperature. VBNC cells can be detected, for example, by using PCR amplification (Gião and Keevil, 2014). As such, an important role is given to the development of new strategies for detecting biofilm formation.

Other novel methods for biofilm detection studies, including metagenomics and metatranscriptomics, can shed light on the complex interactions within a biofilm community (McLean and Kakirde, 2013; Jahid and Sang-Do, 2014; Mukherjee et al., 2016). For example, *S. aureus* subspecies typing was possible using the multiple locus variable number of tandem repeats analysis (MLVA) in samples of food industry products. This method used PCR amplification of diverse *S. aureus* loci which show variable number of tandem repeats (*sdr, clfAclfG, ssp, spa*) and gel electrophoresis to distinguish between detected genotypes. These genotypes showed different lengths in the amplified PCR fragments (Rešková et al., 2014; Coughlan et al., 2016).

However, these common detection methods (at laboratory level) such as agar plating, qRT-PCR or more specific DNA amplifications (Li et al., 2017) are not effective at industrial level, due to the already describe presence of VBNC cells in some biofilms (in the case of agar plating) and to the high cost of reactives and equipments (in the cases of qRT-PCR and other DNA amplifications). Thus, an important role has been given to the development of new strategies to detect biofilm formation in industrial environments, where the practical development of most biofilm on-line monitoring methods is generally based on the introduction of an external perturbation in the system. Then this perturbation can be measured by a suitable device and/or amplified, in order to be converted into a calibrated value. For example, at industrial level, heat transfer and pressure measurements can be used. In the for food and beverage sector, some commercial on-line monitoring sensors have been specially designed, based on thermal pulse analysis. In these sensors, the local thermal conductivity and heat variations due to biofilm formation are measured. These systems are able to detect deposits only a few micrometers thick (Fratamico et al., 2009).

Another common commercial methodology is the measurement of electric signals, which includes electrochemical measurements and capacitance/impedance determinations. Some commercially available monitoring devices, such as BIOX or BIOGEORGE, measure the coupling current between stainless steel and zinc electrodes connected by a resistance. This system has also been used to optimize dosage of antibiofilm compounds in industrial environments (Fratamico et al., 2009).

Finally, another alternative technology is the measurement of vibration signals. The commercial quartz crystal microbalance (QCM) device analyzes the effect that the adhesion of biofilms or other deposits to a quartz crystal causes on the vibration (frequency) of such a surface. The Q-Sense device is a commercial version of QCM devices, and it is able to detect the initial adhesion of bacteria to stainless steel surfaces. The Mechatronic Surface Sensor (MSS) has two transducers attached to the monitored industrial surface. One of them acts as an exciter/actuator and the other one acts as a sensor in order to measure the propagated wave (with or without biofilm on that surface). The MSS device is able to detect biofilm deposits but also to distinguish among these and abiotic deposits (Pereira et al., 2008). An interesting feature for MSS device is that it can be used on stainless steel, copper, PVC, glass and other industrial surface materials, just on the outersurface of the pipe of interest (Fratamico et al., 2009).

**FIGURE 1:**
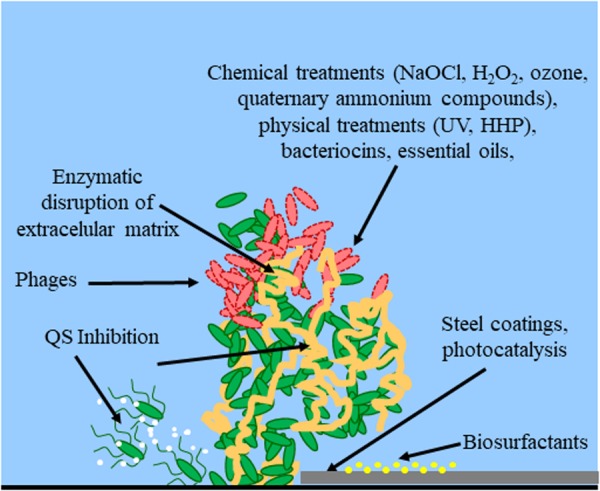
Control methods for biofilm establishment, development and eradication. Red bacterial cells indicate dead cells. White dots indicate QS signals. Yellow dots indicate the treatment of the surface with biosurfactants. The extracellular matrix is indicated in orange. Arrows indicate the site of action for methods targeting bacterial cell integrity (chemical treatments, physical treatments, bacteriocins, essential oils), extracellular matrix (enzymatic disruption), cell-to-cell communication (QS inhibition), or physical properties of the surface (steel coatings, biosurfactants, photocatalysis).

Along with these classical or novel detection methods, new strategies for preventing biofilm formation must be developed that avoid bacteria to build resistance to disinfectants in food processing environments. Various physical methods (such as hot steam, ultrasonication) and chemical compounds (such as sodium hypochlorite, sodium hydroxide solutions, hydrogen peroxide, peracetic acid, etc.) are nowadays used to control biofilm formation in the food industry, including within pipelines and on working surfaces. Depending on the specific industry process, cleaning and disinfection of the whole infrastructure is possible, in order to constantly avoid microbial attachment to pipelines or surfaces. Alternatively, clean-in-place methodologies maintain clean surfaces by spraying or recirculating liquids (Srey et al., 2013). However, as these biofilms are complex communities, their unique characteristics increase the possibility of chemical and physical resistances, making their elimination very difficult in some cases, and favoring their persistence in the industry environment. Therefore, the development of new antibacterial approaches, focused on preventing biofilm formation instead of its elimination is very important in this industrial sector (Gopal et al., 2015). The following sections will describe most important methods for controlling biofilm formation in the food industry, including some novel strategies such as bacteriophages, bacteriocins, quorum sensing inhibitors, essential oils, high hydrostatic pressure and non-thermal plasma (**Table 1** and **Figure 1**).

**Table 1:** Biofilm control methods for their use in the food industry.

Methodology	Examples	Mechanism of action	Reference
Chemical treatments	Sanitizers (NaOCl, peracetic acid, NaOH, H_2_O_2_)	Cell structures oxidation	Rosenberg et al., 2008; Bayoumi et al., 2012; Schmidt, 2012; Bang et al., 2014; Nam et al., 2014; Ban and Kang, 2016; Techaruvichit et al., 2016; Yang et al., 2016; Møretrø et al., 2017
Enzymatic disruption	Cellulases	Extracellular matrix disruption	Wang et al., 2012; Coughlan et al., 2016; Stiefel et al., 2016
	Proteases		Oulahal-Lagsir et al., 2003; Chaignon et al., 2007; Boels, 2011; Huang et al., 2014; Coughlan et al., 2016; Stiefel et al., 2016
	Glycosidases		Boels, 2011; Huang et al., 2014; Coughlan et al., 2016
	DNAses		Coughlan et al., 2016
Steel coatings	Nanoparticles (Ag^2+^, Fe_3_O_4_, TiO_2_, ZnO, CuO, MgO	Alteration of bacterial membrane	Alexander, 2009; Beyth et al., 2015; Rai et al., 2015
	Repelling surfaces (monolayers, hydrogels, modified topography)	Inhibition of bacterial binding	Campoccia et al., 2013; Jindal et al., 2016; Swartjes and Veeregowda, 2016
	Functionalized surfaces (with lisozyme or nisin)	Bactericidal	Sandreschi et al., 2016; Gu et al., 2017
Biosurfactants	Lichenysin	Inhibition of bacterial adhesion	Coronel-León et al., 2016
	Surfactin		Zhang et al., 2017; Zhao et al., 2017
Bacteriophages	P100	Cell lysis	Fister et al., 2016; Iacumin et al., 2016
Bacteriocins	Nisin	Cell membrane alteration	Strempel et al., 2015
QS inhibition	Binding of inhibitors to QS receptors (lactic acid)	Downregulation of adhesion and virulence mechanisms	Rasmussen et al., 2005; Brackman and Coenye, 2015; Coughlan et al., 2015; Amrutha et al., 2017
	Eznymatic degradation of QS signals (paroxonases)		Dong et al., 2001; Yang et al., 2005; Uroz et al., 2008; Koh et al., 2013
	sRNA post-transcriptional control		Perez-Martinez and Haas, 2011
	Inhibition of QS signals biosynthesis		Adonizio et al., 2008; Chung et al., 2011; Zhu et al., 2015; Al-Shabib et al., 2016
	Furanones	Motility inhibition	Keskinen and Annous, 2011; Vestby et al., 2014
Essential oils	Citral	QS inhibition, motility inhibition	Shi et al., 2017
	Carvacrol	Bactericidal	Friedman, 2014
High hydrostatic pressure	H_2_O	Bactericidal (also endospores)	Evelyn and Silva, 2015; Santos et al., 2017
Non-thermal plasma	UV plus O_2_, N_2_, O_3_, H_2_O and He	Bactericidal	Scholtz et al., 2015
Photocatalysis		Bactericidal	Chorianopoulos et al., 2011; Priha et al., 2011; Nica et al., 2017; Ishwarya et al., 2018

### Chemical Treatments

Several concentration-dependent and time-dependent chemical sanitizers can be used as biofilm treatment. The objective is to reduce microbial populations to levels that are safe for humans, a process called sanitization (Schmidt, 2012). Sanitization of food processing equipment is essential for the prevention of cross contamination between batches of food (Bayoumi et al., 2012).

Chlorine-based sanitizers are the most widely used in the food industry, but resistance to chlorine treatments has arisen in some microbes. For example, in *S. enterica*, chlorine resistance was correlated to its cellulose production phenotype. This phenotype depended on the environmental stress conditions found in the food processing plants (Yang et al., 2016). Aqueous ClO_2_ is the most widely used sanitizer in the food industry, although gaseous ClO_2_ has been demonstrated to be more effective against *B. cereus* endospores present in biofilms on food steel surfaces (Nam et al., 2014). In the case of *E. coli* O157:H7 biofilms, aqueous ClO_2_ was more effective than NaOCl (sodium hypochlorite, commonly used at 50–1,000 ppm), especially when a drying step followed the surface treatment at the food factory. Furthermore, this treatment with ClO_2_ increased the sensitivity of *E. coli* cells to other stressors, such as drying (Bang et al., 2014).

*Staphylococcus aureus* and *S. enterica* are common pathogens in raw milk microbiota and can easily form biofilms in dairy factories. NaOCl is an effective chemical for eradicating these biofilms on stainless steel and polypropylene surfaces. However, this disinfectant was unable to eradicate the pathogen *Cronobacter sakazakii* biofilms in the same environments (Bayoumi et al., 2012).

H_2_O_2_ is a potent oxidizing disinfectant, widely use in food industry. It generates free radicals in contact with the biofilm structures, destroying them at concentrations of 0.08–5% without toxic side effects (Srey et al., 2013). Its combination with acetic acid generates peracetic acid, a strong oxidant with pH 2.8, which is used, for example, in water pipes treatment at 0.5%, with high efficacies against *L. monocytogenes* and *S. aureus* populations (Srey et al., 2013).

Ozone is a toxic gas with a potent oxidizing activitiy as well. It destroys different kinds of microorganisms, even biofilms, viruses and protozoans, by breaking down the cellular envelopes (Srey et al., 2013). Its use in dairy industry prevents mold overgrowth on stainless steel structures, powdered formulas and cheeses, for example (Varga and Szigeti, 2016).

Quaternary ammonium compounds (as Metaquats) are widely used as sanitizers in food industry, including biofilms removal. These positively charged water soluble compounds disrupt the bacterial cell membrane, causing bacterial lysis (Jennings et al., 2016). However, some *L. monocytogenes* strains isolated from food environments harbor genes involved in resistance to quaternary ammonium sanitizers (*qacH* and *bcrABC*), which act as pumps for secretion of these compounds. This characteristic can allow them to persist after sanitization procedures. These genes provide growth advantages for bacteria in food manufacturing plants and, therefore, another type of sanitizer or higher concentrations must be used (Møretrø et al., 2017). In this cases, a multi-faceted approach using a combination of different treatments could improve the removal of biofilms formed by these resistant bacteria. For example, a combination of NaOCl, H_2_O_2_, iodophor and benzalkonium chloride with steam heating was able to eliminate biofilms formed by *E. coli* O157:H7, *S. enterica* and *L. monocytogenes*, decreasing both sanitizer concentrations and treatment times (Ban and Kang, 2016).

Another example of acquired resistance to biocides is *Campylobacter jejuni*, a common human pathogen. This bacterium is able to acquire resistance to three common biocides used in the food industry: acetic acid, sodium hypochlorite and trisodium phosphate, after its cultivation in increasing sublethal concentrations. It is also worth noting that this pathogen forms biofilms with distinct structures after its exposure to different biocides. This suggests that the bacterium is able to secrete different extracellular matrixes (exoproteins, exopolysaccharides, amyloid fibers) depending on the chemical environment (Techaruvichit et al., 2016). In such cases, antibiotics covalently linked to carbohydrate carriers (such as chitosan) can be used to overcome these resistances (Zhang et al., 2013).

Other less common sanitizers, such as salicylate-based polyanhydride esters, interfere with the biofilm formation in *S. enterica* at the air-liquid interface. This implies that biofilm formation by this pathogen can be prevented in the initial stages (Rosenberg et al., 2008). The synthetic brominated furanone F202, also an uncommon sanitizer, inhibited *S. enterica* and *E. coli* O103:H2 growth at temperatures used in the food industry, preventing the formation of these biofilms on abiotic surfaces and also targeting the flagellar function of both bacteria. This fact accentuates the potential of furanones as a treatment for eradicating biofilms in the food industry (Vestby et al., 2014). Finally, an experimental short-chain fatty acid formulation resulted in a promising sanitizer against *E. coli* O157:H7 biofilm formation on fresh vegetables (Keskinen and Annous, 2011).

It is interesting to note that decades of sanitizers use in the food industry may be one of the main driving forces with respect to development of antibiotic resistance in bacteria and its dissemination to pathogens. Therefore, development of alternative technologies could be a first step toward reduction of this important health problem worldwide (Capita and Alonso-Calleja, 2013).

### Enzymatic Disruption

Enzymes are considered green countermeasures against biofilm formation since they are biodegradable and have low toxicity. These features make them a powerful tool for biofilm control, and therefore, they are widely used in detergents for food industry applications (Torres et al., 2011; Huang et al., 2014).

The major components of the biofilm structure are comprised of organic macromolecules (mainly proteins and polysaccharides). Therefore, proteases (e.g., serine proteases, proteinase K, pepsin and trypsin) and glycosidases (e.g., amylases, dextranase and pectinase) are always the first option for biofilm removal (Boels, 2011; Huang et al., 2014; Meireles et al., 2016). Pectin methylesterase, for example, is an enzyme capable of reducing biofilm formation in bioreactors. This activity is imperative to the food industry as it can be used as a pretreatment for the various machines and pipes (Torres et al., 2011).

Other enzyme activities such as amylases, cellulases, lyases, glycosidases (such as dispersin B) and DNAses, as part of industrial detergents, are commonly used in the food industry as well to remove biofilms (Coughlan et al., 2016). For example, a treatment involving several cellulases, followed by immersion in a bath with cetyltrimethyl ammonium bromide, produced the complete removal of a biofilm generated by seven *S. enterica* strains isolated from meat processing surfaces (Wang et al., 2016). Alpha-amylase, another example, is effective in degrading *S. aureus* biofilms (Thallinger et al., 2013).

Proteases generally have lower substrate specificities and are therefore more efficient in treating organic-based biofilms (Huang et al., 2014). Once the biofilm matrix is partly degraded by these proteases, it can be completely removed by mechanical treatments and it is more sensitive to the action of sanitizers (Coughlan et al., 2016). For example, a mix of proteolytic enzymes (protease XXIII from *Aspergillus oryzae* and trypsin from porcine pancreas) combined with ultrasonic waves for a duration of 10 s was able to remove 96% of the *E. coli* biofilms on the stainless steel surfaces in a dairy plant (Oulahal-Lagsir et al., 2003). Protease formulations were able to efficiently remove *S. aureus* biofilms on polystyrene surfaces as well, but the removal of other species, such as *P. aeruginosa* biofilms, required mixtures of protease, amylase and cellulase (Stiefel et al., 2016). Subtilisins are widely used in industry to combat biofilm formation by, for example, *P. aeruginosa* or *L. monocytogenes*. These serin proteases are produced by *Bacillus* spp. and degrade adhesins, which are important bacterial attachment proteins to surfaces (such as stainless steel) of to other bacteria. Some new variants are even able to destroy these targets at 90°C temperatures, which enhances their industrial use as ingredients of detergents (Thallinger et al., 2013).

*Staphylococcus aureus* biofilms can be classified into two main groups depending on production levels of poly-*N*-acetylglucosamine (PNAG). The sensitivity of these two types of biofilms was tested against dispersin B, proteinase K, trypsin and pancreatin. In these experiments, dispersin B was an effective agent against biofilms containing high amounts of PNAG, which normally are resistant to proteases. However, the other three proteases were effective against biofilms with low amounts of PNAG. So, a combination of these enzymes could eradicate a heterogeneous *S. aureus* biofilm (Chaignon et al., 2007).

Some pathogens, as *E. coli, L. monocytogenes*, or *S. aureus* secrete exogenous DNA during biofilm formation as well. In these cases, bovine DNase can be added to destroy these multicellular structures (da Silva and De Martinis, 2013). The combination of enzymes with different activities, as well as with other chemical (sanitizers) or physical (ultrasounds) treatments enhanced biofilm removal in different species as *E. coli* or *Bacillus sp* (Meireles et al., 2016). However, the implementation at industrial scale of all these enzymatic alternatives is still hampered due to the high cost of these treatments, mainly due to patent protections.

### Steel Coatings

The poor response of biofilms to conventional control methods highlights the urgent need for alternative antibacterial and antibiofilm agents. One promising approach focuses on nanotechnology agents. The unique properties of nanoparticles (NPs) distinguish them from their bulk chemical counterparts. One such property is their large surface area to volume ratio, which creates a higher number of functional sites and can enhance the influence of NPs on a given microorganism. Since the antibacterial properties of some NPs are mediated mainly by direct contact with the bacterial cell wall and do not require penetration, most bacterial antibiotic resistance mechanisms are not relevant when dealing with NPs (Beyth et al., 2015). Therefore, NPs are less prone to induce bacterial resistance than traditional antibiotics. This favorable property has stimulated extensive research on the antibacterial effects of diverse NPs types, such as carbon-based materials (fullerenes and carbon nanotubes), dendrimers that provide cavities for other molecules, nanocomposites, natural NPs and metal-based NPs, including silver, gold, metal oxides (such as ZnO and CuO) (Rai et al., 2015).

Due to their potent antimicrobial effects, silver compounds have been used since ancient times (Egyptians, Greeks, Romans) to prevent microbial infections associated for example to water consumption (Alexander, 2009; Ebrahiminezhad et al., 2016). Currently, silver NPs are the most widely studied. However, metal oxide NPs are more commonly used within industry. They include iron oxide (Fe_3_O_4_), titanium oxide (TiO_2_), zinc oxide (ZnO), copper oxide (CuO) and magnesium oxide (MgO). These NPs show antimicrobial properties and can be applied in diverse industrial environments. These organic and inorganic NPs can be modified with different atoms, materials or other NPs. The resulting nanocomposites can potentially exhibit improved or new properties that can be exploited for multifunctional applications. As such, these hybrid nanostructure systems represent an area of extensive research (Rai et al., 2015).

Another interesting approach consists in exploiting the effectiveness of different nanocomposite materials toward reducing bacterial adhesiveness. For example, sulfhydryl compounds such as cysteine, dithiothreitol or beta-mercaptoethanol were able to reduce *S. aureus* biofilm formation on polystyrene polymer by inhibiting extracellular matrix biosynthesis genes such as *ica* (Wu et al., 2011).

Bacterial adhesiveness is influenced by multiple chemical and physical properties of the surface, including hydrophobicity, electrical charge and functional groups, all of which can determine the kinetics of bacterial adhesion (Campoccia et al., 2013). Bacteria-repelling surfaces are usually composed of inert materials where the repellent property is provided by self-assembled monolayers, polymer brushes, hydrogel coatings or by manipulating the surface morphology or topography. Alternatively, this repelling activity is conferred by a coating of intrinsically antibacterial and antibiofilm materials (Swartjes and Veeregowda, 2016). One example is the coating of a stainless steel surface with the modified plastic Ni-*P*-polytetrafluoroethylene. This compound was able to reduce biofilm formation by *Geobacillus stearothermophilus* and *Bacillus licheniformis* by two orders of magnitude, in comparison with the control stainless steel surface. It was also effective in preventing milk deposition along the same surface (Jindal et al., 2016).

In line with this latter strategy, functionalized surfaces with polymers including lysozyme in their composition showed antibacterial and antibiofilm properties of great industrial interest: they were able to kill 95, 92, and 94% of *E. coli, S. aureus*, and *C. albicans* biofilms, respectively. These polymers contained mainly insoluble lysozyme manufactured as a flexible film (Huang et al., 2016; Gu et al., 2017).

A final approach to surface treatment is the use of liposomal formulations containing the bacteriocin nisin, or its inclusion in nanofiber membranes of poly-lactic acid and soybean protein. These strategies showed antimicrobial activity against the development of *S. aureus* biofilms on treated working surfaces (Sandreschi et al., 2016).

### Biosurfactants

Biosurfactants are natural compounds, usually of microbial origin, able to modify the hydrophobic characteristics of the bacterial surface. This alters the adhesion properties and binding capacities to any given surface. One of these molecules is lichenysin, a cyclic non-ribosomal lipopeptide produced by *B. licheniformis*. Food industry surfaces can be treated with this biosurfactant, which can diminish the binding of microbes such as MRSA (50% adhesion inhibition at 8.3 μg/mL), *C. albicans* (at 17.2 μg/mL), *Y. enterocolitica* (at 16.1 μg/mL) or *C. jejuni* (at 188.5 μg/mL) (Coronel-León et al., 2016).

Fengycin, iturin, and surfactin are similar lipopeptides produced by *B. amyloliquefaciens* (and *B. subtilis* in the case of surfactin). All these compounds act on the surface of the corresponding target microbe, altering its binding capacity by decreasing surface tension. These molecules insert themselves into the microbial cell membranes, or chelating cations. This effect alters the membrane permeability, eventually disrupting it and causing cell swelling and death (Zhang et al., 2017; Zhao et al., 2017).

### Bacteriophages

The antimicrobial activity carried out by bacteriophages is innocuous to humans, animals, and plants due to their specific killing of prokaryotic cells. As a result, phage therapy is currently an attractive alternative to antibiotics. Their use as antibiofilm agents is a promising approach and has already lead to commercial applications. For example, one application based on *Listeria* phage P100 (under the commercial name Listex P100) was produced to eliminate biofilms present in processed meat products and on factory working surfaces, and has already been authorized in the United States by the Department of Agriculture with the status of GRAS biological agent (Fister et al., 2016; Iacumin et al., 2016). Other pathogen species have been targeted with commercial bacteriophages products, as for example *S. enterica* or *E. coli* (Salmofresh^TM^ and ScoShield^TM^, respectively) (Gutiérrez et al., 2016).

The primary limitation of phage treatments is their ability to access and target bacterial cells inside the biofilm. This limitation exists due to the intricate biofilm structure and the presence of extracellular material, which acts as a physical obstacle to phage diffusion. However, some phages possess exopolysaccharide depolymerases, an excellent solution for this diffusion problem (Pires et al., 2016). The presence of these enzymes enhances the phage invasion and dispersion process through the biofilm under treatment (Parasion et al., 2014).

Endolysins and virion-associated peptidoglycan hydrolases have also been assessed as biofilm removal agents because they easily penetrate the biofilms (Shen et al., 2013; Gutiérrez et al., 2014). Other factors can influence phage treatments, such as the synergy/antagonism between phages and conventional sanitizers or the temperatures commonly used in the food industry, which may be not optimal for phages. Another relevant issue is the effect of these phages on mixed biofilms formed by different species, which is common on food industry surfaces (such as communities of *B. cereus* with *L. monocytogenes*, etc.) (Gutiérrez et al., 2016).

In summary, phages and phage-derived proteins are highly effective in biofilm removal at lab scale, though some commercial examples do exist. However, more research is needed before their complete implementation as part of the standard cleaning processes in the food industry (Gutiérrez et al., 2016). Specifically, phage safety (for humans) and the absence of an environmental impact (in the case of engineered phages) must be addressed. Other issues that must be addressed before their full implementation in the food industry are the technical problems related to their manufacturing, such as their scaling up during propagation, as well as phage purification and, in EU, the EFSA regulatory frane (Nobrega et al., 2015; Gutiérrez et al., 2016).

### Bacteriocins

The use of bacteriocins in the food industry is useful to prevent biofilm formation on different surfaces. These antimicrobial agents can extend the expiration date of a given food as well, protects against alterations during refrigeration, lowers food spoilage, prevents the transmission of foodborne pathogens, diminishes chemical preservative concentrations and reduces the number of temperature treatments.

For example, nisin, a 34 amino acid polycyclic peptide isolated from *Lactococcus lactis* (Mattick and Hirsch, 1947), has been approved for its antimicrobial activity since 1969 (WHO, World Health Organization) and 1988 (FDA, USA Food and Drug Administration) because the consumption of this peptide is safe for animals and humans. So far, nisin remains the only FDA approved bacteriocin in the food industry (Strempel et al., 2015). Used as spray on surfaces used for food manufacturing, nisin was able to prevent adhesion and biofilm formation by *L. monocytogenes* (García-Almendárez et al., 2008).

Other bacteriocins have been extensively investigated for preventing bacterial colonization, especially those produced by GRAS (Generally Recognized as Safe) microorganisms, such as lactic acid bacteria. Some of these novel bacteriocins are pediocins (active against *L. monocytogenes* and produced by *Enterococcus* spp.), lactocins (active against *Brochothrix thermosphacta* and produced by *Lactococcus* spp.) and garvicin (produced by *Lactococcus garvieae* and active against pathogenic strains of this bacterium) (Castellano et al., 2017). Their use does not represent a risk with respect to animal tissues, and therefore, similarly to current nisin, their commercial implementation should not represent a serious issue.

### Quorum Sensing Inhibition

Different signaling pathways are required for bacterial biofilm formation and antimicrobial resistance development. These include the exchange of small organic molecules or proteins as well as the transmission of electrical signals (Liu et al., 2015; Prindle et al., 2015; Kim et al., 2016). Among these signaling pathways, quorum sensing (QS) and cyclic di-GMP (cGMP) signaling are the best characterized. QS is a widely distributed intercellular signaling mechanism. It is used by bacteria to regulate gene expression in response to high environmental concentrations of small diffusible signaling molecules (acyl homoserine lactones, peptides and the autoinducer-2). These QS regulated mechanisms include genes involved in biofilm formation (Parsek and Greenberg, 2005; Yang and Givskov, 2015).

In a similar way, high intracellular cGMP content triggers the biosynthesis of extracellular polymeric substances and it reduces bacterial motility, facilitating biofilm formation (Römling et al., 2005). Many small molecules can inhibit cGMP biosynthesis, such as the terpenoid saponin (Ohana et al., 1998), nitric oxide generating compounds (Barraud et al., 2009), azathioprine (Antoniani et al., 2013), or sRNAs (Pérez-Martínez and Haas, 2011).

Based on these data, an effective strategy for eradicating food-associated bacterial biofilms is to prevent their formation by using QS inhibition (Coughlan et al., 2015, 2016). QS has been described, for example, in *L. monocytogenes* as an important factor regulating biofilm development and maturation (da Silva and De Martinis, 2013). QS inhibitors (QSI) have been proposed as a new generation of antimicrobial agents since they act primarily by quenching the QS system mediators (QQ, quorum quenching). Unlike bactericidal strategies, compounds targeting QS and biofilm formation cause less selection pressure and, therefore, do not develop resistance to the inhibitory compound (Brackman and Coenye, 2015).

Different strategies used to interfere with bacterial QS based on the inhibition of cell-to-cell communication include competitive binding of inhibitors to the QS receptors (Rasmussen et al., 2005), enzymatic degradation of QS signals (Dong et al., 2001), post-transcriptional control of QS genes via sRNAs (Perez-Martinez and Haas, 2011) and inhibition of QS signals biosynthesis (Chung et al., 2011).

Another strategy involves the use of paraoxonases. Paraoxonases are a type of QQ enzymes. These enzymes are isolated from mammalian sera and are useful in blocking QS since they cause the hydrolysis of the lactone ring of *N*-acyl homoserine lactone (AHL), an important signal molecule in the case of *P. aeruginosa* (Yang et al., 2005). These enzymes have also been isolated from root-associated fungi and various plants (Uroz et al., 2008; Koh et al., 2013). AHL communication system is the target of diverse plant compounds with QSI activities as well, as the halogenated furanones from the red alga *Delisea pulchra*. In a similar way, disulphide compounds from garlic and rosmarinic acid from rosemarin act as QSI and prevent biofilm formation in *P. aeruginosa* (Koh et al., 2013).

Organic acids are another type of QQ in the food industry. For example, 2% of lactic acid produced a 1 log reduction in *E. coli* and *Salmonella* spp. CFUs. Citric acid and acetic acid have also QS potential but to a lesser extent than lactic acid (Amrutha et al., 2017).

New QSI can oftentimes be obtained from natural sources. For example, extracts from grapefruit and grapefruit juice, rich in furocoumarins, showed QS inhibition in *Vibrio harveyi* as well as in *E. coli* O157:H7, *S. enterica* and *P. aeruginosa*. Also, extracts from different North American plant species (*Bucidabuceras, Callistemon viminalis*, and *Conocarpus erectus*) were effective against *P. aeruginosa* biofilm formation. This effect was due to QS inhibition at the level of its regulators Vfr and GacA (Adonizio et al., 2008). Moreover, green tea polyphenols diminished protease activity and trimethylamine production in *Shewanella baltica*, inhibiting its biofilm formation. The QSI activity of these polyphenols was associated with the inhibitory activity of epigallocatechin gallate and this could be used to prevent against seafood spoilage caused by biofilms (Zhu et al., 2015). Furthermore, *Nigella sativa* seed extract binding to zinc NPs interfered with motility and matrix production during the initial attachment and also during the biofilm maturation by *E. coli, L. monocytogenes*, and *P. aeruginosa* (Al-Shabib et al., 2016).

### Essential Oils

Several compounds derived from plants demonstrated antibiofilm properties. One useful advantage related to the use of these compounds as antibiofilm agents is the positive perception consumers have of them, in contrast to chemical synthesis disinfectants, especially in food industry applications. Plant-based essential oils are primarily a species-specific complex mixture of monoterpenoids (such as borneol, camphor, carvacrol, eucalyptol, limonene, pinene, thujone), sesquiterpenoids (such as caryophyllene, humulene) and flavonoids (such as cinnamaldehyde and other phenolic acids) (Raffaella et al., 2017).

Some of these essential oils show antibiofilm properties. For example, a 24 h old *S. aureus* biofilm on steel was reduced from 10^7^ CFU/mL to 10^3^ CFU/mL using a *Cinnamomum cassia* essential oil microemulsion (very rich in cinnamaldehyde) at 2.5% in TSB medium for a 90 min period. A similar CFU reduction was obtained with a 5% essential oil microemulsion of *Salvia officinalis*, which is rich in thujone, camphor and pinene (Raffaella et al., 2017).

Biofilms from three important Gram-negative pathogens, *S. enterica, E. coli*, and *P. aeruginosa*, were also reduced up to 80% using extracts at 50 μg/mL from the Asian medicinal plants *Holarrhena antidysenterica* and *Andrographis paniculata*. In this case, the effect was due to the damage that the essential oil cinnamaldehyde component caused to the bacterial cell membrane (Masák et al., 2014; Tanwar et al., 2016; Thakur et al., 2016).

*Cronobacter sakazakii* is also a biofilm producer strain and citral (the main component of lemongrass oil) was proven as an anti-adhesion and antibiofilm compound. Citral decreased virulence factors and reduced the biosynthesis of flagella in this pathogen. It also interfered with this pathogen’s cell-to-cell signaling, diminishing its virulence and its biofilm formation (Shi et al., 2017).

The huge chemical diversity of plant-based essential oils allows for the development of *à la carte* antibiofilm preparations against various pathogens. Essential oils from *Origanum vulgare* show anti-adhesion properties, which are crucial in preventing food spoilage and foodborne pathogens. For example, carvacrol, a monoterpene from oregano essential oil, can be used in vinegars, fresh juices, minced meat and other foods, where it is effective in inhibiting *Clostridium perfringens* and *S. enterica* development. This terpenoid was evenly mixed at 1% in plastic films intended for food applications, inhibiting bacterial growth and biofilm development (Friedman, 2014). In particular, Sicilian oregano (*O. vulgare* ssp. *hirtum*) essential oil could potentially prevent or eradicate biofilms in the food industry (Schillaci et al., 2013). According to Desai et al. (2012), oregano and thyme oils also showed a highly efficient eradication of diverse strains and serotypes of *L. monocytogenes* biofilms on polystyrene and stainless steel surfaces. Carvacrol is also very effective against *L. monocytogenes* and *S. aureus* biofilms (Giaouris et al., 2014). Similarly, thymol, another monoterpenoid from *Thymus vulgaris* oil, was able to reduce *L. monocytogenes* biofilms at diverse temperatures (37°C, 25°C, 4°C), as well as *S. enterica* biofilms (Marchese et al., 2016). All these examples show the important antibiofilm activity of essential oil components on diverse pathogens biofilms, although some of these essential oils have been defined by EFSA as potential irritants to skin and other human organs, and therefore are considered as bioactive plant extracts (European Food Safety Authority, 2014).

### High Hydrostatic Pressure

High hydrostatic pressure (HHP, 300–900 MPa) is able to destroy or inactivate vegetative bacterial cells. However, this technology is not effective in the case of endospores (such as those in the case of *B. cereus*), unless a pretreatment is carried out at lower pressures (300–400 MPa) in order to allow germination of existing spores (Evelyn and Silva, 2015). Anyway, some non-germinating spores could remain in the food matrix after HHP treatments, and therefore, at industrial level, HHP is usually combined with thermal treatments (50°C to 100°C), or in some cases with essential oil components (Luu-Thi et al., 2015). One important advantage of HHP treatments is that they do not alter the organoleptic and nutritional properties of the food matrixes (taste, vitamins, etc.), a great adavantage with respect to high temperature methods (Santos et al., 2017).

### Non-thermal Plasma

Non-thermal plasma is a partially ionized gas with low temperature and interesting antimicrobial properties. It is produced at atmospheric pressure by mixing UV light with oxigen, nitrogen, ozone, and water and helium, under an electrical discharge. It is able to destroy bacterial biofilms of Gram-negative (*Pseudomonas* spp., *S. enterica*) or Gram-positive (*Bacillus* spp.) species in just 10 min, However, its use is still restricted to some laboratory applications, due to its high cost (Scholtz et al., 2015).

### Photocatalysis

Diverse types of nanoparticles show photocatalytic properties, where the absorption of an specific wavelength is used for generating (accelerating) a chemical reaction, including destruction of microbial cells, generally due to reactive oxygen species (ROS) generation (Nica et al., 2017). In this sense, TiO_2_ NPs, containing 1% Fe and N, structured as a thin layer on a polystyrene surface, have demonstrated inactivation of bacterial cells (*E. coli, Enterococcus faecalis, P. aeruginosa, S. aureus*) after sun light exposure. When exposed to visible, and specially to UV light, these NPs also showed antibiofilm activity in the case of *E. coli, P. aeruginosa* and *S. aureus*, which inhibition values in the order of 2–32 μg/mL (Nica et al., 2017). Similar TiO_2_ NPs, although containing Ag instead of Fe and N, have been tested successfully on stainless steel surfaces (coupons) in order to prevent bacterial biofilm formation, a method of potential application in beverage industry pipelines (Priha et al., 2011). This coating strategy has been more effective in the case of Gram-negative bacteria (such as *Pseudomonas fluorescens*) than in the case of Gram-positive ones (such as *Lactobacillus paracasei*) (Priha et al., 2011).

ZnO NPs generated with the help of *Ulva lactuca* aqueous extract have been also described as excellent photocatalysts, absorbing light at 325 nm wavelength. This NPs are able to generate ROS in contact with bacterial cells, causing 80% reductions in biofilms formed by *Bacillus pumilus, B. licheniformis, E. coli* or *Proteus vulgaris* (Ishwarya et al., 2018).

Photocatalytic NPs have demonstrated strong antibiofilm effects against important human pathogens as well. For example, 10-days-old *L. monocytogenes* biofilms, generated after incubation at 16°C on stainless steel or glass surfaces, have shown reductions of 3 log CFU/cm^2^ after 180 min and 120 min irradiation with 395 nm wavelength, respectively (Chorianopoulos et al., 2011). This novel technique demonstrates the feasibility of biofilm removal using self-disinfecting modified surfaces in industrial environments, where stainless steel and glass surfaces are common.

## Conclusion

Food industry biofilms constitute a serious economic and health issue. On the one hand, the existence of biofilms along food manufacturing surfaces can lead to financial losses as a result of corrosion on the metal surfaces by some bacteria, requiring the replacement of these parts. Furthermore, some bacterial species, such as *Pseudomonas* spp. and *Bacillus* spp., secrete many different proteolytic and lipolytic enzymes that can generate unpleasant odors (rancid) and tastes (bitter). In these instances, the affected manufacturing batches must be removed and destroyed. These disruptions may not only represent a significant financial loss for the company, but may also potentially damage their brand with respect to their competitors.

On the other hand, and of more vital concern, biofilm formation in food factories represents a crucial public health issue. These biofilms may contain bacterial (and sometimes fungal) species known to be pathogenic in healthy individuals or at times only targetting the immunocompromised (such as organ transplant recipients, oncology or HIV patients, etc.). These pathogens can cause food intoxications (*B. cereus, S. aureus*) and, in some cases, gastroenteritis (*E. coli, S. enterica*) and systemic diseases (*E. coli* O157:H7, *L. monocytogenes*).

Food companies traditionally rely on cost-effective chemical methods, such as sodium hydroxide or sodium hypochlorite solutions, as well as physical methods, such as hot water steam, ozone or mechanical removal techniques, for the elimination or prevention of biofilm development along the factory pipes and surfaces. However, biofilm development on some food industry structures or packaging methods cannot be controlled with this cost-effective methods. Therefore, the use of more approaches for biofilm removal, such as enzymatic disruption (using detergents containing proteases, glysosidases or DNase), steel surface modification (by coating with silver, cupper or zinc nanoparticles, or by using the novel antibiofilm polymers with lysozyme or bacteriocins), or biosurfactants such as lichenysin (added to industrial detergents) is common at industrial level to control biofilms.

Novel technologies have been developed during the last years as well, although their implementation at industry level requires always the corresponding authorization from the health authorities, and their use is in some cases limited to some few commercial examples such as the use of bacteriophages targeting specific biofilm-forming bacterial species (for example *L. monocytogenes* in the case of the phage P100, restricted mainly to meat factories and products) or the use of bacteriocins for destruction (lysis) of the biofilm cells in dairy products (such us nisin, pediocins, lactocins or garvicin).

Finally, novel promising options arise in some food industry environments and products, and will be implemented in the coming years. Some examples here are the quorum sensing inhibitors, such as paraoxonases (which degrade quorum sensing signal molecules) or rosmarinic acid, lactic acid, citric acid, furocoumarins and several plant flavonoids. Another example are plant essential oils, where bioactive compounds (plant secondary metabolites) such as cinnamaldehyde, citral, carvacrol or thymol are able to reduce bacterial biofilms on different surfaces. The list of novel technologies includes also high hydrostatic pressure (up to 900 MPa) combined with thermal treatments (50–100°C), a method which is interesting for endospores removal; and the highly expensive non-thermal plasma treatments. All these novel techniques promise an optimistic future for controlling biofilms formation in the food industry.

## Author Contributions

SG contributed to *B. cereus* and steel coatings section. CG-G contributed to *E. coli*, chemical treatments and bacteriocins sections. EM contributed to *L. monocytogenes* and QS section. CV contributed to *S. enterica* and biosurfactants, bacteriophages sections. FL contributed to *S. aureus*, enzymatic disruption, essential oils, HHP, NTP and photocatalysis sections.

## Conflict of Interest Statement

The authors declare that the research was conducted in the absence of any commercial or financial relationships that could be construed as a potential conflict of interest.
